# Detection of Deregulated Modules Using Deregulatory Linked Path

**DOI:** 10.1371/journal.pone.0070412

**Published:** 2013-07-24

**Authors:** Yuxuan Hu, Lin Gao, Kai Shi, David K. Y. Chiu

**Affiliations:** 1 School of Computer Science and Technology, Xidian University, Xi’an, Shaanxi, China; 2 College of Science, Guilin University of Technology, Guilin, Guangxi, China; 3 School of Computer Science, University of Guelph, Guelph, Ontario, Canada; Semmelweis University, Hungary

## Abstract

The identification of deregulated modules (such as induced by oncogenes) is a crucial step for exploring the pathogenic process of complex diseases. Most of the existing methods focus on deregulation of genes rather than the links of the path among them. In this study, we emphasize on the detection of deregulated links, and develop a novel and effective regulatory path-based approach in finding deregulated modules. Observing that a regulatory pathway between two genes might involve in multiple rather than a single path, we identify condition-specific core regulatory path (CCRP) to detect the significant deregulation of regulatory links. Using time-series gene expression, we define the regulatory strength within each gene pair based on statistical dependence analysis. The CCRPs in regulatory networks can then be identified using the shortest path algorithm. Finally, we derive the deregulated modules by integrating the differential edges (as deregulated links) of the CCRPs between the case and the control group. To demonstrate the effectiveness of our approach, we apply the method to expression data associated with different states of Human Epidermal Growth Factor Receptor 2 (HER2). The experimental results show that the genes as well as the links in the deregulated modules are significantly enriched in multiple KEGG pathways and GO biological processes, most of which can be validated to suffer from impact of this oncogene based on previous studies. Additionally, we find the regulatory mechanism associated with the crucial gene SNAI1 significantly deregulated resulting from the activation of HER2. Hence, our method provides not only a strategy for detecting the deregulated links in regulatory networks, but also a way to identify concerning deregulated modules, thus contributing to the target selection of edgetic drugs.

## Introduction

Revealing the mechanisms of oncogenes is a major challenge in the study of complex diseases, while detecting deregulated modules induced by oncogenes brings a solution for such issue. Many previous studies devote to identifying deregulated or mutated pathways and subnetworks, which provide a way to elucidate the molecular mechanisms in the pathogenic process. Ideker et al. [1] present a network-based approach to search for the ‘active’ connected subnetworks by integrating protein-protein, protein-DNA interaction network with gene expression. The genes considered in these subnetworks are significantly differentially expressed. Based on this seminal work, Nacu et al. [2] improve the scoring function and algorithm considering the correlation among genes. The results are more biologically interpretable. Ulitsky et al. [3] give a method for identifying deregulated connected subnetworks in protein-protein interaction network by analyzing the deregulation of genes using clinical expression profiles. Based on gene set enrichment analysis, a dynamic programming algorithm is presented for finding significantly deregulated paths in tumor cells by Keller et al. [4]. Recently, Backes and coworkers [5] propose an integer linear programming method for identifying deregulated modules and molecular key players, which are responsible for deregulation. Liu et al. [6] propose a method for detecting deregulated pathways from derived pathway interaction network. They formulate the detection as a feature selection problem based on defining activity scores for each pathway.

Besides these methods for detecting deregulated modules, there exist several approaches for identifying diverse characteristic subnetworks, such as response subnetworks after drug treatment [7], functional modules [8], signal transduction networks [9], and optimally discriminative subnetworks for classification [10]. Most of these network-based approaches can be modeled as an optimization problem by defining adequate scoring functions for nodes (genes) [1]–[5], [8], [10]. Especially for detecting deregulated modules, a typical strategy is that fold change or t-statistical test of gene expression is used as the measurement for deregulation of genes under two different cell states. Optimal connected subnetworks are then identified using different searching algorithms [1], [3], [5]. As comparison, Vandin et al. [11] define an influence measure between pairs of genes rather than their scores to identify mutated subnetworks using a diffusion process. To our knowledge, most of the existing methods are not originally designed for directed networks [1]–[3], [11]. They focus on the deregulation of genes rather than the links among them [1]–[5]. Thus, the regulatory information carried by directed networks is not well utilized. However, as the development of edgetic drugs (or drugs targeting network edges) is on the rise, little attention has been focused on the gene regulatory networks in this field [12]. We propose here a promising new direction to study the deregulation of links in the regulatory network, and provide candidate targets for the development of edgetic drugs.

In this paper, we develop a novel and effective regulatory path-based approach for the detection of deregulated modules when induced by oncogenes. Different from existing works, we not only consider the deregulation of the genes themselves by constructing a differentially regulated network, but also provide a strategy for detecting the deregulated links in regulatory networks. Observing that a regulatory pathway between two genes might involve in multiple rather than a single path [13], we design a method to identify the condition-specific core regulatory path (CCRP) from the upstream gene to the downstream gene. The regulatory effect of the CCRPs plays an important role in the regulatory mechanism. From the same upstream to the downstream gene, the CCRP may be switched due to the state change of the proto-oncogenes, reflecting the significant deregulation of the regulatory links. Our method consists mainly of three key steps: 1) First, given the time-series gene expression datasets of the case and the control group, the regulatory strength within each regulator-target gene pair is measured. For accuracy, we use the log marginal likelihood scoring method, taking into account the transcriptional time lags [14]. 2) Second, the shortest path from the upstream to the downstream gene is used to depict the selected CCRP. As attempting to detect significant switch of the CCRPs, we select deregulated pivot genes as seeds to analyze their associated links. Considering the impact of oncogenes on the regulatory mechanism associated with known crucial genes, such genes can be taken as seeds. 3) Finally, we derive the deregulated modules by integrating the differential edges of the CCRPs in the regulatory subsystems centered on the seed genes between the case and the control group.

To evaluate our approach, we experiment on the detection of deregulated modules induced by the Human Epidermal Growth Factor Receptor 2 (or HER2) oncogene, which is expressed in approximately 25% of human breast cancers. In our work, the KEGG human regulatory network [5] and two time-series gene expression datasets are integrated and analyzed to generate the deregulated modules. The two datasets are respectively measured from the breast epithelial cell lines with insulin-independent transformed phenotype (resulting from the HER2 oncogene overexpression) and with insulin-dependent non-transformed phenotype (or HER2 as a proto-oncogene). Using the same network and the expression datasets, we make comparisons between our method and the approach presented by Backes et al. [5], which is primarily designed for directed networks. In summary, we focus on the detection of deregulated links by analyzing the CCRPs in regulatory networks. The effectiveness of our method is demonstrated by the functional enrichment analysis and the literature evidence. Since evenly spaced time-series gene expression data with large number of time points are still scarce, we focus on analyzing this group of expression datasets in this paper, but further studies on other types of data will extend our understanding of this approach in the future.

## Materials and Methods

### Overview

**Figure 1:**
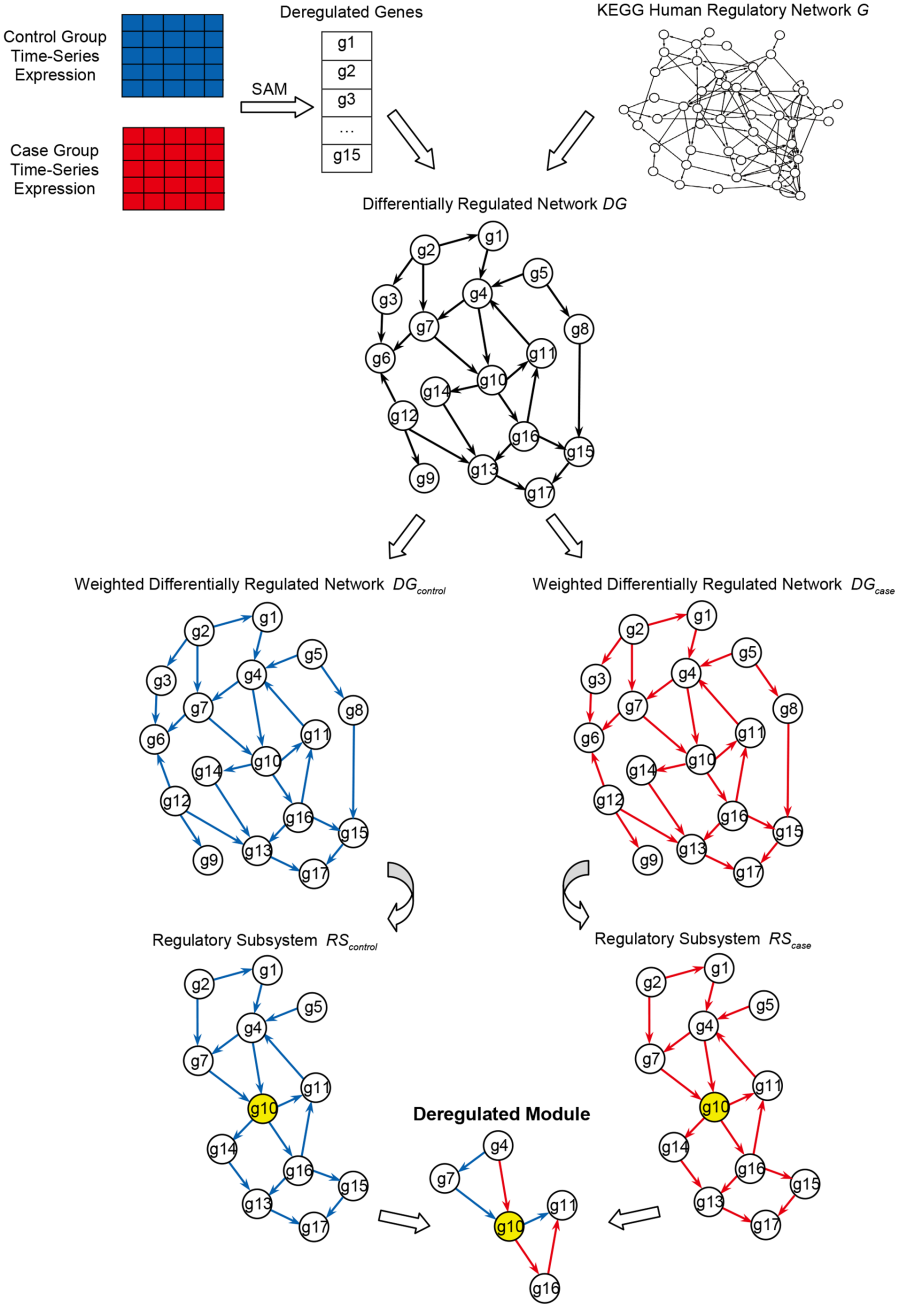
Workflow of our approach. The figure shows the workflow of our approach. Given the time-series gene expression datasets of the case and the control group, 15 deregulated genes are selected to map to the nodes of KEGG human regulatory network *G*. Allowing for the integrity of links among genes, we construct the differentially regulated network *DG* consisting of 17 nodes and 25 directed edges. For each regulator-target gene pair, we compute the regulatory strength of the control (colored by blue) and the case (colored by red) and the two weighted differentially regulated network 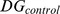 and 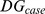 are constructed. Centered on the seed gene ‘g10’, which is colored by yellow, the two regulatory subsystems 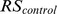 and 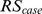 are extracted. The deregulated module is derived by integrating the differential edges of the CCRPs. Here, the CCRP from ‘g4’ to ‘g11’ is switched. In the control group, the CCRP is ‘g4→g7→g10→g11’. While in the case group, it is ‘g4→g10→g16→g11’.

As a brief introduction to our approach, our method considers a gene regulatory network using two time-series expression datasets corresponding to the case (when the proto-oncogene is activated) and the control group (when the proto-oncogene is in a normal state). We screen out the deregulated genes in constructing a differentially regulated network. The regulatory strength of the case and the control group is then computed as weights to this network. Centered on each seed gene, two regulatory subsystems are constructed. Finally, we derive deregulated modules by integrating the differential edges of the CCRPs, which are identified using a shortest path algorithm. The workflow of our approach is shown in Figure 1.

### Construction of Differentially Regulated Network

We identify differentially expressed genes using SAM (Significance Analysis of Microarrays) [15] as deregulated genes in constructing a differentially regulated network. Using time-series expression datasets of the case and the control group, a differentially expressed score of each gene is calculated (using SAM). We screen out deregulated genes using a cutoff which is determined using statistical analysis. These genes are then mapped to the nodes of the KEGG human regulatory network (denoted as *G*) constructed using the method by Backes et al. [5]. The procedure integrates all the KEGG regulatory pathways including the KEGG cancer pathways into a network, which consists of 2004 nodes (genes) and 10147 directed edges (as regulatory links). Allowing for the integrity of the links among genes, the mapping is that if one of the associated nodes of an edge in the network *G* can be found among the deregulated genes, the edge will be reserved. Additionally, all the reserved nodes should have their corresponding expression used for the computation of regulatory strength. Otherwise, the edge will be removed. In the following, we denote this network as the differentially regulated network 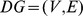, where 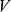 is the set of nodes, and 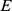 is the set of directed edges.

### Scoring for Regulatory Strength

For identification of the CCRPs, we define the regulatory strength within each regulator-target gene pair as the statistically dependent degree of a target on its regulator.

We use the log marginal likelihood scoring as the regulatory strength between a regulator and its target, considering the transcriptional time lag. The scoring scheme is provided by Zou et al. [14], which requires evenly spaced time-series gene expression as input. The transcriptional time lag between each regulator and its target is estimated using the gene expression of the control group. Then, with the time lag taken into account, the two log marginal likelihood scores of each gene pair are computed under the case and the control group, respectively. The score reflects the fitting degree of a gene pair expression on their link. Therefore, a high score indicates that the target is strongly dependent on its regulator, reflecting a high regulatory strength within the gene pair.

**Figure 2:**
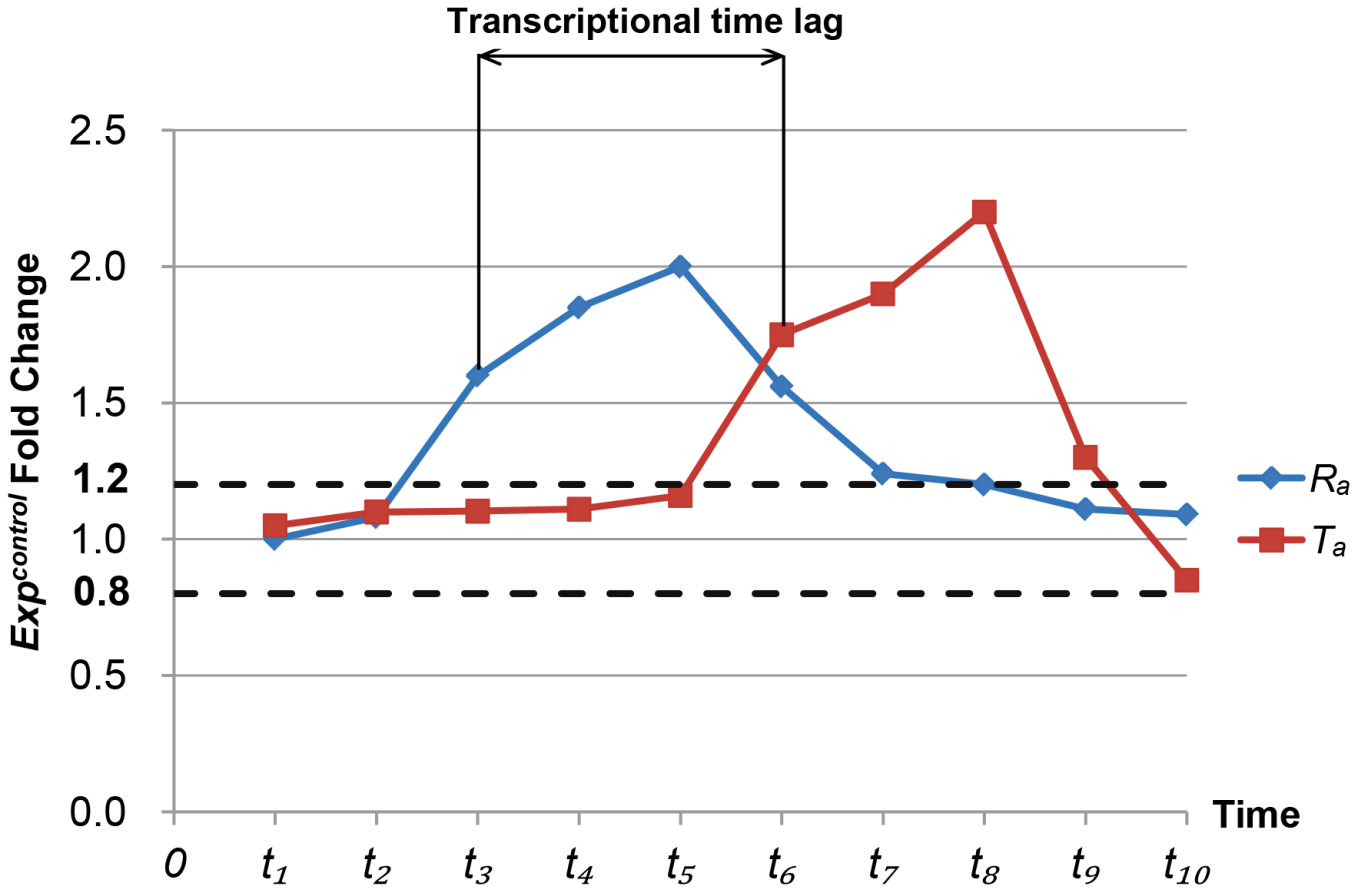
Estimation of transcriptional time lag. The figure shows the estimation of transcriptional time lag. In this example, with respect to the 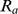-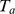 gene pair, the time points of the initial changes in their expression of the control group are 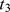 and 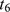, respectively. Thus, the transcriptional time lag is 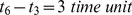.

The scoring method is explained as follows. Estimating the transcriptional time lag of each gene pair, we determine the time points of the initial changes in their expression of the control group. Here, the expression of the case group is not used for the estimation due to the deregulation effect induced by the oncogenes. Based on the work of Zou et al. [14], we compute the fold changes of the gene expression at each time point compared to the baseline expression. A 1.2 fold (up regulation) or 0.8 fold (down regulation) is used as a cutoff. For example, with respect to the *i-th* gene pair 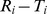 in the network *DG*, the time points of their initial changes 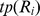 and 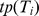 are determined according to the following equations:
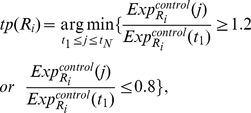
(1)

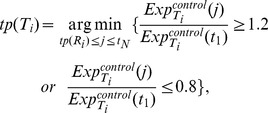
(2)where 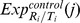 denotes the gene expression of 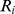 or 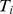 at time point 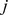 in the control group. The time-series includes *N* evenly spaced time points 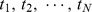. Since most transcriptional regulators exhibit either an earlier or simultaneous change in the expression when compared to their targets [16], we only consider the time from 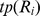 to 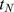 when computing the initial change time point of target 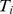. The difference, 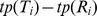, is defined as the transcriptional time lag. As an example, we use the expression of a regulator 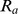 and its target 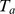 and is shown in Figure 2.

**Figure 3:**
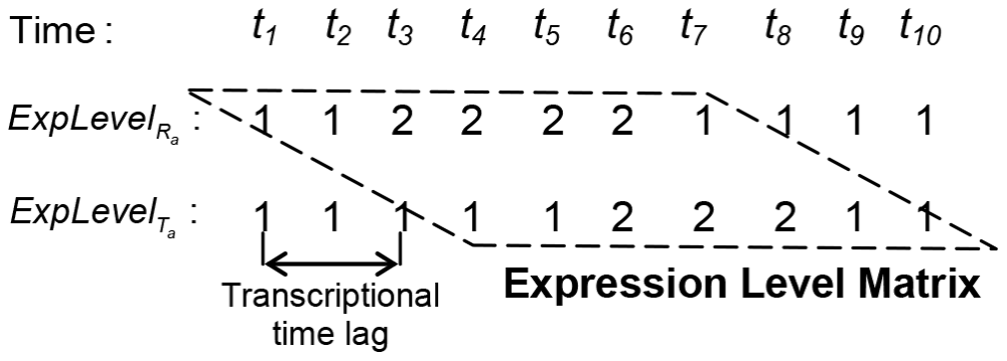
Construction of expression level matrix. As is shown in Figure 2, the transcriptional time lag between 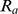 and 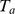 is 3 *time unit*. After discretizing the expression of the gene pair, we organize 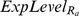 and 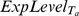
into the expression level matrix, where the expression level of 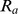 at time point 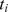
is aligned with the expression level of 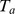 at time point 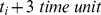.

Next, we discretize the expression of each gene into two levels as a simplification for the statistical analysis. More levels can be used if necessary. For each gene, if the expression at a time point is lower than the average expression, the level at this time point will be discretized as ‘1’. Otherwise, it will be ‘2’. Based on Zou’s scoring [14], we utilize the discretized expression levels of each gene pair with its estimated transcriptional time lag to construct an expression level matrix. The example is shown in Figure 3.

Finally, with the expression level matrices as inputs, we use the Murphy’s Bayes Net Toolbox (BNT) (https://code.google.com/p/bnt/) to compute the log marginal likelihood scores as regulatory strength. In order to facilitate subsequent computation, we define the weight of each edge in the *DG* by
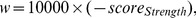
(3)where 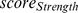 refers to the related regulatory strength (i.e. the log marginal likelihood score). Thus the two weighted differentially regulated networks 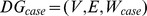 and 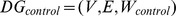 are constructed.

### Construction of Regulatory Subsystem Centered on a Seed Gene

Given the regulatory strength, the relative importance of multiple regulatory paths between two genes can be ranked. We use the shortest path from the upstream gene to the downstream gene in depicting the CCRP. Since the pivot genes identified based on betweenness centrality have strong ability to control the CCRPs among other genes, the deregulation of such genes may cause significant switch of the CCRPs. For detecting the significantly deregulated links, it is important to select deregulated pivot genes as seeds.

Specifically, the betweenness centrality [17] of each node in the network 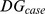 and 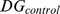 is computed as:
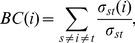
(4)where 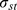 is the total number of shortest paths from node 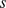 to node 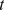 and 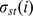 is the number of those paths which pass through node 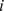. Thus, 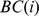 depicts the ability of gene 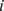 to control the CCRPs among other genes. In other words, if the gene 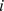 gets a high betweenness centrality, it is considered to be a pivot, playing an important role in maintaining the normal regulatory mechanism. After computing the betweenness centrality of all the nodes in both 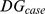 and 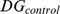, we select the top ranking genes (as deregulated pivots) as our seeds based on their statistical distribution. From the perspective of crucial genes identified as causing diseases (or deregulation), such genes can be taken as seeds to evaluate the impact of oncogenes on their associated regulatory network, which could be a very important issue in understanding the regulatory mechanism that biologists concern.

We extract the upstream and the downstream subnetworks of a seed gene from 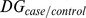, whose combination is denoted as a regulatory subsystem. Centered on each seed gene, two regulatory subsystems 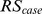 and 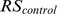 are thus constructed.

### Detection of the Deregulated Modules

The CCRPs in the 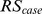 and 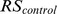 are identified using the shortest path algorithm [18]. From the same upstream to the downstream gene, the differential edges of CCRPs between the case and the control group are identified as the deregulated links. With respect to different gene pairs, these links may have the same parts. It is clear that the more the same parts that exist, the more stable and significant the deregulated module will be. We then integrate the deregulated links of all the upstream-downstream gene pairs into a subnetwork denoted as the deregulated module. It is worth noting that there is no deregulated link for some gene pairs, indicating that the associated regulatory mechanism does not suffer significant impact from the oncogenes. For different seed genes, multiple deregulated modules can be derived.

### Over-Representation Analysis on Deregulation of Genes

To evaluate our method, we perform the over-representation analysis (ORA) on the deregulated modules with GeneTrail [19]. The genes of a module and the network *G* are used as test set and reference set, respectively. The significance p-values are computed using the False Discovery Rate (FDR) adjustment method proposed by Benjamini and Hochberg [20].

### Network Ontology Analysis from the Deregulation of Regulatory Links

We also perform network ontology analysis (NOA) [21] from the deregulated links. NOA is a powerful tool to capture the function changes caused by the switch of the CCRPs, which is induced by the activity of the oncogenes. In the deregulated module, the edges that belong to the CCRPs in 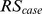 and the 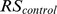 are used as test set, respectively. All the edges in the network *G* are used as reference set. We can then observe the changes of enriched GO biological processes between the control and the case group.

## Results

To evaluate our proposed method, we analyze the time-series gene expression of the breast epithelial cells with two different metabolic phenotypes due to the state change of HER2. The amplification or overexpression of this oncogene plays an important role in the pathogenesis of multiple human cancers.

### Time-Series Expression Datasets

Two time-series gene expression datasets are respectively measured after the treatment of the HER2-specific inhibitor CP724, 714 for the breast epithelial cells with insulin-independent transformed phenotype (denoted as the “case” group) and with non-transformed phenotype (denoted as the “control” group) [22]. Such treatment blocks HER2 kinase activity, which facilitates to detect the differentially regulatory effects of HER2 on other genes and their links between the activated state and the normal state. It is the differential regulation that reflects the deregulation induced by the oncogene. In the datasets, each time-series includes 16 time points with even time lag and the expression profiles are retrieved from NCBI Gene Expression Omnibus (GEO) with the accession number of GSE23137 and GSE23138. The gene expression profile is obtained using the Illumina humanRef-8 v2.0 expression BeadChip with 20589 features corresponding to 18190 genes. The signal intensity is log2 transformed and normalized using quantile normalization with IlluminaGUI in R. Subsequently, we apply our method to these expression datasets to identify deregulated modules in the regulatory network *G*.

### Deregulated Modules

We evaluate the selection of the seed genes using two different ways. First, deregulated pivot genes are selected as the seeds to detect significant switch of the CCRPs induced by the HER2 oncogene. Second, in order to study the impact of HER2 on the regulatory mechanism associated with known crucial genes, we select snai1 homolog1 (SNAI1), currently thought to be involved in tumour invasion [23], as seed to analyze its associated CCRPs.

**Figure 4:**
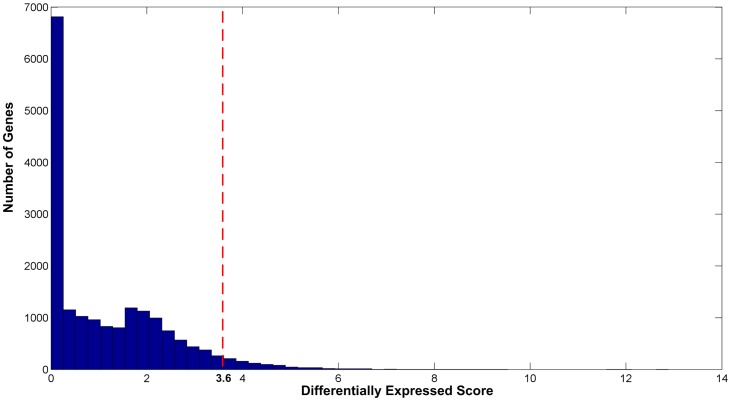
Statistical distribution of differentially expressed scores of all the genes in the microarray. SAM is used to compute the differentially expressed score of each gene. And the score of 3.6 (*P*<0.05) is considered as a cutoff to select deregulated genes.

1) Impact on the CCRPs associated with the deregulated pivot genes. Since the seed genes to be selected are of strong control ability and significantly deregulated feature, we use SAM with the differentially expressed score of 3.6 (*P*<0.05) as a cutoff to screen out deregulated genes. Here, the cutoff is determined based on the statistical distribution of differential scores of all the genes in the microarray, which is shown in Figure 4. Then, the directed network *DG* consisting of 654 nodes and 1015 edges is constructed. After computing the regulatory strength, we use MatlabBGL (http://dgleich.github.io/matlab-bgl/index.html), a Matlab graph package, to compute the betweenness centrality of all the genes in the 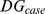 and 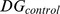. When the top ranking genes are selected, the value of 1000 (*P*<0.05) is used as a cutoff. Thus, we obtain 20 different seed genes (including the first 19 genes of the case group and first 20 genes of the control group), which are listed in Table 1. We then derive 20 deregulated modules from the corresponding regulatory subsystems. In particular, we find none of these deregulated modules has a unique structure such that eight, four and three of the 20 modules have the same structures, respectively. The remaining modules are either the subgraphs of the aforementioned modules or have similar structures with each other. This observation indicates that the identified deregulated modules are stable and significant. We denote the union of these modules as the integrated deregulated module.

**Table 1:** List of seed genes for detecting significant switch of CCRPs.

Entrez Gene ID	Gene symbol	Betweenness centrality
22800	RRAS2	6511
4301	MLLT4	5357
3675	ITGA3	5089 (5139)
998	CDC42	4351 (4353)
4067	LYN	3411
6714	SRC	3089
5582	PRKCG	3079 (3420)
5908	RAP1B	3048
81607	PVRL4	3039 (2968)
1445	CSK	2970
5921	RASA1	2723
3676	ITGA4	2562
8835	SOCS2	1970
5781	PTPN11	1913
9020	MAP3K14	1507
5058	PAK1	1488
6777	STAT5B	1313 (1307)
10451	VAV3	1258
2697	GJA1	1180
7297	TYK2	1008 (966)

The table lists 20 seed genes including first 20 genes of the control group and first 19 genes of the case group based on betweenness centrality computed by MatlabBGL. In the third column, when betweenness centrality of a gene is different between the two groups, the corresponding unit has two values. Otherwise, only one value is shown.

**Figure 5:**
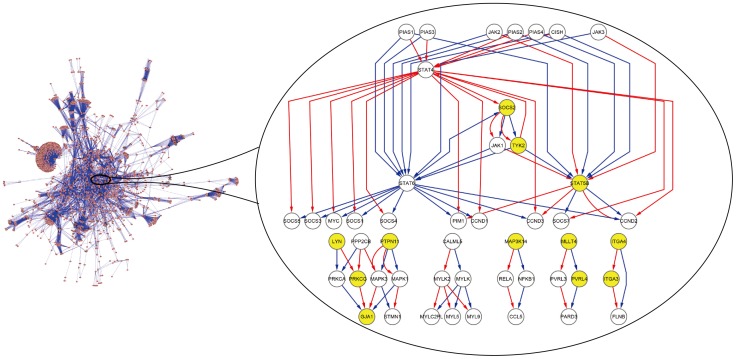
Integrated deregulated module. The figure shows the integrated deregulated module consisting of 49 gene nodes and 86 directed edges, which include both 43 edges belonging respectively to the control (colored by blue) and the case group (colored by red). The seed genes are colored by yellow. Note that not all the 20 seed genes appear in the deregulated module.

Using the visualization software Cytoscape [24], Figure 5 shows the integrated deregulated module consisting of 49 nodes and 86 directed edges. When performing ORA on this module, we find that many KEGG pathways associated with cancers are significantly enriched. Remarkably, most of the enriched pathways are verified to suffer significant impact from the HER2 oncogene (for examples see [22], [25]–[35]). Ten of the strongly validated enriched KEGG pathways with their significance adjusted p-values are listed in Table 2. The remaining enriched pathways include at least five genes of the integrated deregulated module (Table S1). When performing NOA on this deregulated module with the 43 edges belonging to the control group as test set, we obtain a group of enriched GO biological processes. Similarly except for setting another 43 regulatory links belonging to the case group as test set, another result is obtained. Comparing the two enrichment results (Table 3), we find that the functions are mainly enriched in the ‘Regulation of biological process’, ‘Metabolic process’, and ‘Cellular process’ in the control group. In the case group, besides the regulation of different metabolic processes, the functions are significantly enriched in the ‘Signal transmission via phosphorylation event’, ‘Intracellular protein kinase cascade’, and ‘JAK-STAT cascade’. (Note in the ‘Discussion’ section, we will further elaborate the enriched KEGG pathways and the GO biological processes and compare our method with the approach presented by Backes et al. [5].).

**Table 2:** Significantly enriched KEGG pathways with respect to the integrated deregulated module.

Enriched KEGG pathway	Expected number of genes	Observed number of genes	p-value (FDR adjusted)
Jak-STAT signaling pathway	3.12	24	1.2416e-15
Ubiquitin mediated proteolysis	1.11	6	0.00273782
Chemokine signaling pathway	3.25	10	0.00329205
Pathways in cancer	6.85	15	0.00545622
Glioma	1.43	6	0.00705588
Non-small cell lung cancer	1.18	5	0.0122739
Adherens junction	1.81	6	0.0168058
ErbB signaling pathway	1.94	6	0.0215711
Tight junction	2.97	7	0.0371006
Insulin signaling pathway	3.02	7	0.0388454

The table lists the results of ORA on the integrated deregulated module. Ten of the strongly validated significantly enriched KEGG pathways are presented. Their significance p-values are calculated using the FDR adjustment method.

**Table 3:** Significantly enriched GO biological processes with respect to the integrated deregulated module.

Control group	Case Group
Negative regulation of biological process (GO: 0048519)	Regulation of macromolecule metabolic process (GO: 0060255)
Regulation of developmental process (GO: 0050793)	Signal transmission via phosphorylation event (GO: 0023014)
Response to chemical stimulus (GO: 0042221)	Intracellular protein kinase cascade (GO: 0007243)
Response to organic substance (GO: 0010033)	Regulation of metabolic process (GO: 0019222)
Positive regulation of macromolecule metabolic process (GO: 0010604)	Regulation of primary metabolic process (GO: 0080090)
Positive regulation of metabolic process (GO: 0009893)	Intracellular signaling pathway (GO: 0023034)
Regulation of macromolecule metabolic process (GO: 0060255)	JAK-STAT cascade (GO: 0007259)
Positive regulation of cellular process (GO: 0048522)	Positive regulation of cell cycle (GO: 0045787)
Positive regulation of cellular metabolic process (GO: 0031325)	Regulation of cellular metabolic process (GO: 0031323)
Positive regulation of biological process (GO: 0048518)	Cytokine-mediated signaling pathway (GO: 0019221)

The table lists the results of NOA on the integrated deregulated module. All the significantly enriched GO biological processes in the control and case group are presented, respectively.

**Figure 6:**
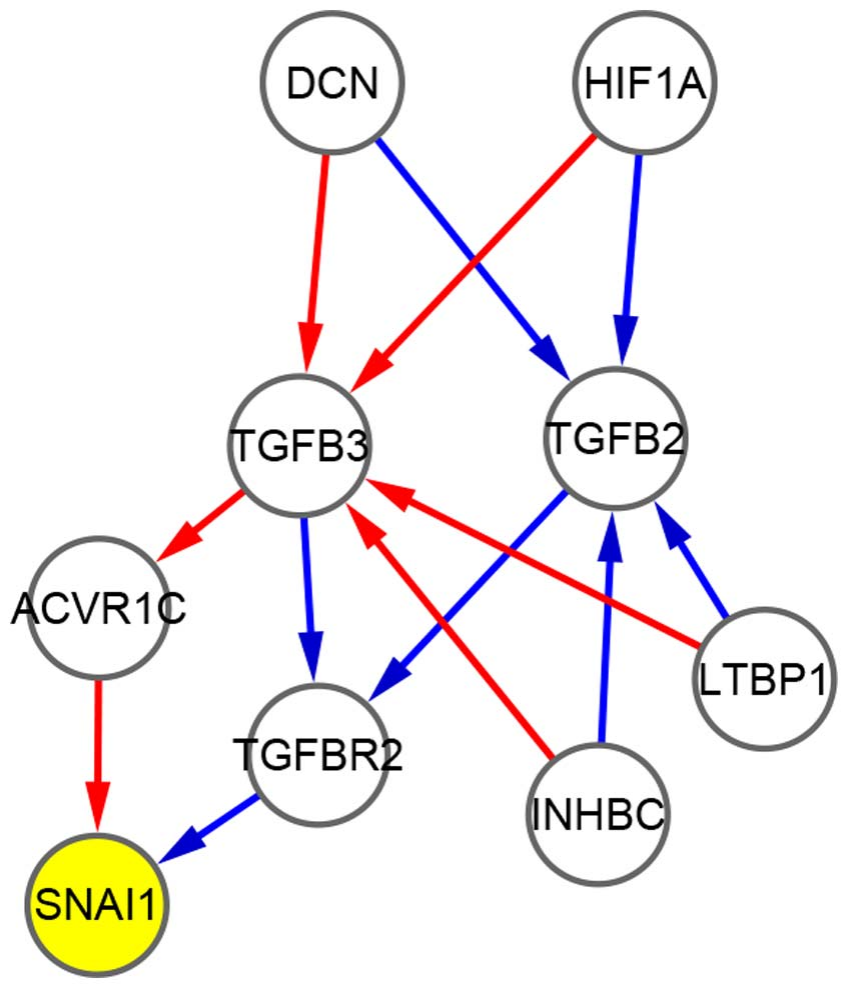
SNAI1 associated deregulated module. The figure shows the SNAI1 associated deregulated module consisting of 9 gene nodes and 13 directed edges. This includes 7 and 6 edges belonging respectively to the control (colored by blue) and the case group (colored by red). The seed gene SNAI1 is colored by yellow.

2) Influence on the CCRPs associated with known crucial genes. Transcriptional factor SNAI1 regulates the epithelial-mesenchymal transition (EMT), a process where cancer cells attain fibroblastic features and thus invade the surrounding structures [36]–[38]. Here, we focus on the deregulation of the SNAI1 associated regulatory mechanism. Since the crucial gene SNAI1 is involved in sparse links in the network, strictly high SAM cutoff will change the structure substantially of its associated CCRPs. We lower the cutoff to construct a network *DG* with relatively large size (1284 nodes and 3792 edges). With SNAI1 as the seed gene, we derive a deregulated module consisting of 9 nodes and 13 edges, shown in Figure 6. When we perform ORA on this deregulated module, the result shows that many cancers related KEGG pathways are significantly enriched, including ‘Colorectal cancer’, ‘Pancreatic cancer’, and ‘Renal cell carcinoma’. Moreover, we find that ‘TGF-beta signaling pathway’ is the most significantly deregulated (*P*<4.26e-8). All the enriched pathways are given in Table S2. We also perform NOA to analyze the result. Making comparisons between the two groups of enriched GO biological processes (Table 4), we observe that the functions are significantly enriched in the morphogenesis related processes and different receptor signaling pathways in the control group. While in the case group, the functions are significantly enriched in the process of ‘regulation of gene-specific transcription’ and ‘response to hypoxia’. Detailed elucidation on the enriched KEGG pathways and GO biological processes is presented in the next section.

**Table 4:** Significantly enriched GO biological processes with respect to the SNAI1 associated deregulated module.

Control group	Case Group
Anatomical structure morphogenesis (GO: 0009653)	Wound healing (GO: 0042060)
Organ morphogenesis (GO: 0009887)	Regulation of localization (GO: 0032879)
Positive regulation of cell differentiation (GO: 0045597)	Positive regulation of gene-specific transcription (GO: 0043193)
Transforming growth factor beta receptor signaling pathway (GO: 0007179)	Response to chemical stimulus (GO: 0042221)
Positive regulation of developmental process (GO:0051094)	Regulation of gene-specific transcription (GO: 0032583)
Organ development (GO: 0048513)	Developmental process (GO: 0032502)
Transmembrane receptor protein serine/threonine kinase signalingpathway (GO: 0007178)	Regulation of secretion (GO: 0051046)
Regulation of cell differentiation (GO: 0045595)	Response to hypoxia (GO: 0001666)
Regulation of epithelial cell migration (GO: 0010632)	Response to oxygen levels (GO: 0070482)
Positive regulation of epithelial cell migration (GO: 0010634)	Aging (GO: 0007568)

The table lists the results of NOA on the SNAI1 associated deregulated module. All the significantly enriched GO biological processes in the control and case group are presented, respectively.

## Discussion

In this work, we propose a novel method for detecting deregulated modules based on the regulatory path. Most of the existing methods use optimization algorithms to search connected subnetworks based on the deregulatory scores of genes. On the other hand, the deregulation of the regulatory links is of much importance in the detection, with intuitive interpretation for edgetic drugs. To compare our method with previous approaches, we apply the method of Backes et al. [5] to the same time-series expression datasets, and integrate the same network in the analysis. Based on their work, we set the size of deregulated modules ranging from 10 to 25 nodes. Combining these 16 optimal modules, we obtain a stable union deregulated module consisting of 33 nodes. We perform ORA with the genes of this deregulated module as test set and the genes of the network *G* as reference set. Compared with the enrichment results of our method, we find that the KEGG pathway of ‘Ubiquitin mediated proteolysis’, ‘Glioma’, and ‘Prostate cancer’ are significantly deregulated in both results. These pathways can be confirmed to be affected by the HER2 oncogene [25]–[27]. It is reported that HER2 overexpression causes the down-regulation of p27 by accelerating the ubiquitin-mediated degradation process of p27, which is an important prognostic marker in many types of cancers [25]. All the enriched KEGG pathways with respect to the union deregulated module generated by Backes’ method [5] are given in Table S3. However in our results, 24 genes in the integrated deregulated module are most significantly enriched in the ‘Jak-STAT signaling pathway’ (*P*<1.25e-15), whose deregulation can contribute directly and indirectly to tumorigenesis [39]. Such significant result is in agreement with the observation that the HER2 oncogene induces JAK-STAT signaling [28]. This deregulated pathway is closely associated with the insulin receptor signaling, which is known to be significantly influenced by the activation of HER2 [22]. Moreover, Li et al. [29] show that in the breast cancer tissues, HER2 enhances the expression of chemokine receptor CXCR4 which can mediate the movement of cancer cells. Such influence may lead to significant deregulation of the chemokine signaling pathway, in which the genes in our results are enriched. Consistent with the observation that overexpression of the HER2 oncogene transforms MCF10A cells into an insulin-independent phenotype [22], we also find ‘ErbB signaling pathway’ and ‘Insulin signaling pathway’ both significantly enriched in the integrated deregulated module. Furthermore, the pathway of ‘Adherens junction’ and ‘Tight junction’ are also significantly deregulated in our results. This can be confirmed as well since Carraway et al. [30] indicate that HER2 oncogene disrupts tight junctions or adherens junctions in order to dissolve interepithelial cell interactions, leading to a loss of cell polarity and the initiation of invasion. Similar conclusion is also reported by Muthuswamy et al. [31].

Different from Backes’ method [5], we can identify the deregulated links by detecting the switch of the CCRPs between the control and the case group. NOA can capture the dynamic changes of enriched functions for the CCRPs. According to the results shown in Table 3, we find that the functions in the control group are not only enriched in the regulation of biological and different metabolic processes, but also significantly enriched in the process of ‘response to chemical stimulus’ (*P*<8.9e-9), which is consistent with the treatment of HER2-specific inhibitor on the cell lines. However, the process of ‘signal transmission via phosphorylation event’ and ‘intracellular protein kinase cascade’ are significantly enriched (*P*<1.2e-7) in the case group, which is in line with similar observation that there is an overall increase in phosphorylation of many pathway proteins associated with cell migration in human mammary epithelial cells due to HER2 overexpression [40]. Based on the finding that cytokines modulate glucose transport and increase the expression of the glucose transporter GLUT1 [41], which facilitates glucose transport in insulin-independent manner, we consider that the deregulated process of ‘cytokine-mediated signaling pathway’ is associated with the cell phenotype of insulin-independent glucose uptake. Additionally, the process of ‘positive regulation of cell cycle’ is also significantly enriched in the case group, which can be confirmed by Harari et al. [42]. Harari et al. observe that HER2 overexpression can lead to dysregulation of the homeostatic machinery of the cell cycle. Furthermore, consistent with the enrichment results of ORA, we also find the process of ‘JAK-STAT cascade’ significantly deregulated (*P*<4.6e-6) in the results of NOA.

The integrated deregulated module is the result by selecting seed genes from the control perspective. In our work, SNAI1, due to its major role in tumor progression, is also selected to be the seed gene. From previous works, Wilson et al. reveal that the overexpression of HER2 alters components of the TGF-beta signaling pathway [43]. Yamanaka et al. find HER2 frequently overexpressing in human pancreatic carcinoma [35]. Co-overexpression of EGFR and HER2 is detected in renal cell carcinoma (RCC) by Stumm et al. [44]. Moreover, the activation of HER2 is detected in colorectal cancers [45]–[47]. In agreement with all these observations, we find that the KEGG pathway of ‘TGF-beta signaling pathway’ (*P*<4.26e-08), ‘Colorectal cancer’ (*P*<7.76e-03), ‘Pancreatic cancer’ (*P*<7.76e-03), and ‘Renal cell carcinoma’ (*P*<7.76e-03) are all significantly enriched in our SNAI1 associated deregulated module according to the results of ORA.

In addition, analyzing the results of NOA (Table 4), we find that the significantly enriched functions in the case group are transformed into the process of ‘regulation of gene-specific transcription’ (*P*<0.02), ‘response to hypoxia’ (*P*<0.025), and ‘response to oxygen levels’ (*P*<0.027), when compared with the enriched functions in the control group. Moreover, Hypoxia-Inducible Factor 1A (HIF-1A) is also a member of the SNAI1 associated deregulated module. Such observations are in line with the results of existing studies. Laughner et al. [48] demonstrate that HER2 oncogene signaling increases the synthesis rate of HIF-1A, which is a “master” gene controlling the hypoxic response and sensitive to the oxygen level. HIF-1A, a transcription factor, can promote the transcription of its multiple specific target genes including glucose transporters and enzymes involved in glycolysis. HIF-1A also enhances the activity of these proteins which plays an important role in maintaining the high rate of glucose uptake [49]. Therefore, increased expression of HIF-1A induced by the HER2 oncogene may contribute partly to the cell phenotype transforming into insulin-independent glucose uptake. Based on these analyses, we can conclude that the SNAI1 associated regulatory mechanism is significantly affected by the HER2 oncogene.

In summary, we have developed a novel and effective regulatory path-based approach for identifying deregulated modules. Furthermore, we apply successfully to the time-series gene expression associated with HER2. Compared with existing methods, our approach is designed primarily for directed regulatory networks, and the regulatory information is well utilized by estimating the regulatory strength within the gene pairs. Outperforming the method by Backes et al. [5], more KEGG pathways are significantly enriched in our results that can be validated by previous studies. Moreover, we not only consider the deregulation of the genes themselves, but also the detection of the deregulated links. Interestingly, even though we do not devise to search connected subnetworks by optimal algorithms [1]–[3], [5], integrating the deregulated links (differential edges of the CCRPs) can often construct a connected subnetwork which indicates a close association among different deregulated pathways. It is noted that functional enrichment analysis and the literature evidence both demonstrate the effectiveness of our method. Our method not only can be applied to gene expression with two stages (e.g. the case and control), it can also be applied to expression with multiple stages. Thus dynamic change of deregulation in the progression of diseases can be detected if a sufficiently large number of time points with even time lag dataset and the appropriate selection of SAM cutoff are available. As a whole, our method provides not only a novel strategy for the detection of deregulated links forming a network, it also identifies concerning deregulated modules, thus contributing significantly to the target selection for the development of edgetic drugs.

## Supporting Information

Table S1 All the significantly enriched KEGG pathways with respect to the integrated deregulated module. The table lists the results of ORA on the integrated deregulated module. All the enriched KEGG pathways which include at least five genes of the integrated deregulated module are presented. And their significance p-values are calculated using the FDR adjustment method.
(DOC)

Table S2 All the significantly enriched KEGG pathways with respect to the SNAI1 associated deregulated module. The table lists the results of ORA on the SNAI1 associated deregulated module. The significance p-values are calculated using the FDR adjustment method.
(DOC)

Table S3 All the significantly enriched KEGG pathways with respect to the union deregulated module generated by Backes’ method. The table lists the results of ORA on the union deregulated module generated by Backes’ method [5]. The significance p-values are calculated using the FDR adjustment method.
(DOC)
