# Supplementary material for: Detection of Deregulated Modules Using Deregulatory Linked Path
Source: PLoS One. 2013 Jul 24;8(7):e70412. doi: 10.1371/journal.pone.0070412 (PMC3722188; doi:10.1371/journal.pone.0070412)
Supplement: Table S1 — All the significantly enriched KEGG pathways with respect to the integrated deregulated module. The table lists the results of ORA on the integrated deregulated module. All the enriched KEGG pathways which include at least five genes of the integrated deregulated module are presented. And their significance p-values are calculated using the FDR adjustment method. (DOC) [file pone.0070412.s001.doc]

## Table S1 All the significantly enriched KEGG pathways with respect to the integrated deregulated module.

| **Enriched KEGG pathway** | **Expected number of genes** | **Observed number of genes** | **p-value (FDR adjusted)** |
| --- | --- | --- | --- |
| Jak-STAT signaling pathway | 3.12 | 24 | 1.2416e-15 |
| Leishmaniasis | 0.91 | 7 | 0.000382676 |
| Acute myeloid leukemia | 1.38 | 8 | 0.000568509 |
| Chronic myeloid leukemia | 1.54 | 8 | 0.000568509 |
| Focal adhesion | 4.66 | 14 | 0.000568509 |
| Small cell lung cancer | 1.91 | 9 | 0.000568509 |
| Type 2 diabetes mellitus | 0.81 | 6 | 0.000568509 |
| Epithelial cell signaling in Helicobacter pylori infection | 1.01 | 6 | 0.00180799 |
| Ubiquitin mediated proteolysis | 1.11 | 6 | 0.00273782 |
| Vascular smooth muscle contraction | 2.04 | 8 | 0.00275727 |
| Chemokine signaling pathway | 3.25 | 10 | 0.00329205 |
| NOD-like receptor signaling pathway | 0.81 | 5 | 0.00329205 |
| Pathways in cancer | 6.85 | 15 | 0.00545622 |
| Glioma | 1.43 | 6 | 0.00705588 |
| Long-term depression | 1.49 | 6 | 0.00787012 |
| Pancreatic cancer | 1.51 | 6 | 0.00804282 |
| Gastric acid secretion | 1.11 | 5 | 0.00973752 |
| Non-small cell lung cancer | 1.18 | 5 | 0.0122739 |
| Adherens junction | 1.81 | 6 | 0.0168058 |
| ErbB signaling pathway | 1.94 | 6 | 0.0215711 |
| Leukocyte transendothelial migration | 2.54 | 7 | 0.0215711 |
| Adipocytokine signaling pathway | 1.43 | 5 | 0.0226992 |
| Chagas disease | 2.09 | 6 | 0.0271881 |
| B cell receptor signaling pathway | 1.59 | 5 | 0.0311995 |
| Fc gamma R-mediated phagocytosis | 1.61 | 5 | 0.0318847 |
| Long-term potentiation | 1.64 | 5 | 0.0325947 |
| Tight junction | 2.97 | 7 | 0.0371006 |
| Insulin signaling pathway | 3.02 | 7 | 0.0388454 |
| Prostate cancer | 1.7871 | 5 | 0.0411558 |

The table lists the results of ORA on the integrated deregulated module. All the enriched KEGG pathways which include at least five genes of the integrated deregulated module are presented. And their significance p-values are calculated using the FDR adjustment method.
