# Supplementary material for: Detection of Deregulated Modules Using Deregulatory Linked Path
Source: PLoS One. 2013 Jul 24;8(7):e70412. doi: 10.1371/journal.pone.0070412 (PMC3722188; doi:10.1371/journal.pone.0070412)
Supplement: Table S2 — All the significantly enriched KEGG pathways with respect to the SNAI1 associated deregulated module. The table lists the results of ORA on the SNAI1 associated deregulated module. The significance p-values are calculated using the FDR adjustment method. (DOC) [file pone.0070412.s002.doc]

## Table S2 All the significantly enriched KEGG pathways with respect to the SNAI1 associated deregulated module.

| **Enriched KEGG pathway** | **Expected number of genes** | **Observed number of genes** | **p-value (FDR adjusted)** |
| --- | --- | --- | --- |
| TGF-beta signaling pathway | 0.34 | 7 | 4.25937e-08 |
| Chronic myeloid leukemia | 0.29 | 3 | 0.00775666 |
| Colorectal cancer | 0.26 | 3 | 0.00775666 |
| Pancreatic cancer | 0.28 | 3 | 0.00775666 |
| Renal cell carcinoma | 0.28 | 3 | 0.00775666 |
| Cytokine-cytokine receptor interaction | 0.77 | 4 | 0.011242 |
| Endocytosis | 0.37 | 3 | 0.011242 |
| Chagas disease | 0.39 | 3 | 0.0117378 |
| Malaria | 0.14 | 2 | 0.0151948 |
| Leishmaniasis | 0.17 | 2 | 0.0195157 |
| Hypertrophic cardiomyopathy (HCM) | 0.18 | 2 | 0.020714 |
| Dilated cardiomyopathy | 0.23 | 2 | 0.0282714 |
| Pathways in cancer | 1.28 | 4 | 0.0370423 |

The table lists the results of ORA on the SNAI1 associated deregulated module. The significance p-values are calculated using the FDR adjustment method.
