# Supplementary material for: Detection of Deregulated Modules Using Deregulatory Linked Path
Source: PLoS One. 2013 Jul 24;8(7):e70412. doi: 10.1371/journal.pone.0070412 (PMC3722188; doi:10.1371/journal.pone.0070412)
Supplement: Table S3 — All the significantly enriched KEGG pathways with respect to the union deregulated module generated by Backes’ method. The table lists the results of ORA on the union deregulated module generated by Backes’ method [5]. The significance p-values are calculated using the FDR adjustment method. (DOC) [file pone.0070412.s003.doc]

## Table S3 All the significantly enriched KEGG pathways with respect to the union deregulated module generated by Backes’ method.

| **Enriched KEGG pathway** | **Expected number of genes** | **Observed number of genes** | **p-value (FDR adjusted)** |
| --- | --- | --- | --- |
| p53 signaling pathway | 0.99 | 25 | 3.81618e-33 |
| Cell cycle | 1.40 | 8 | 0.000770281 |
| Glioma | 0.93 | 6 | 0.00236674 |
| Pyrimidine metabolism | 0.03 | 2 | 0.00236674 |
| Glutathione metabolism | 0.05 | 2 | 0.00562253 |
| Purine metabolism | 0.28 | 3 | 0.013989 |
| Melanoma | 1.04 | 5 | 0.0166282 |
| Prostate cancer | 1.15 | 5 | 0.0203745 |
| Ubiquitin mediated proteolysis | 0.72 | 4 | 0.0203745 |
| Bladder cancer | 0.49 | 3 | 0.0434299 |

The table lists the results of ORA on the union deregulated module generated by Backes’ method [5]. The significance p-values are calculated using the FDR adjustment method.
